# It’s Time to Go Quantum in Medicine

**DOI:** 10.3390/jcm12134506

**Published:** 2023-07-05

**Authors:** Joseph Bisiani, Adith Anugu, Srinivas Pentyala

**Affiliations:** 1 Departments of Anesthesiology, Renaissance School of Medicine, Stony Brook University, Stony Brook, NY 11794, USA; joseph.bisiani@stonybrook.edu (J.B.); adith.anugu@stonybrook.edu (A.A.); 2 Departments of Orthopaedics, Renaissance School of Medicine, Stony Brook University, Stony Brook, NY 11794, USA; 3 Departments of Urology, Renaissance School of Medicine, Stony Brook University, Stony Brook, NY 11794, USA; 4 Departments of Health Sciences, Renaissance School of Medicine, Stony Brook University, Stony Brook, NY 11794, USA; 5 Departments of Physiology, Renaissance School of Medicine, Stony Brook University, Stony Brook, NY 11794, USA

**Keywords:** quantum mechanics, medicine, diagnosis, mind, DNA, telomere

## Abstract

As the field of medicine grows and expands, new scientific developments hold great promise for improving quality of care, clinical research, and the diagnosis and treatment of diseases. Quantum physics is a promising field that intersects with medicine much more than originally understood. In terms of diagnosing different diseases, incorporating quantum mechanics into the study of medicine can allow for efficient diagnosis before symptoms even arise in a patient. Applying theory-based mathematical structures that describe neuron transmission throughout the brain and mind on a quantum scale can help us to better understand neurological diseases in patients. Quantum theory can even give plausible explanations for subtle DNA changes and even telomere reduction in patients who develop cancer. Utilizing quantum theory in the field of medicine can help in understanding and applying treatments for a multitude of different diseases, such as Alzheimer’s disease or diverse types of cancer, and even expand upon efficient and reliable diagnosis in clinical settings. Quantum physics is a pertinent advancing field that may have a significant impact on expanding medical care and treatment in the near future. In this review, the application of quantum physics in medicine is discussed.

## 1. Introduction

The field of medicine is unique in that different areas of the world employ different ideologies and have different approaches to treat, cure, and prevent disease amongst their populations. The actual practice of medicine has been around for centuries, but as civilizations grew and expansion across the world occurred, new cultures arose, and consequently new viewpoints and belief systems came into existence. In turn, the way medicine has been approached by different cultures has been unique to those respective societies. In addition to this, as education expanded and technological innovations continued to develop, new processes, advancements, and breakthroughs in the field of science came about, and the field of medicine inherently changed as well. Medicine is so amazing in that it uses the field of science to help treat individuals for a myriad of different diseases and health-related issues. When people think about medicine in the modern twenty-first century, some fields that come to mind are biology and chemistry, which makes sense. Biology is defined as the structure of living organisms, and medicine is all about treating the human body. Chemistry studies reactions between different molecules and hence is one of the foundational subjects for helping develop drugs and different treatments for a multitude of diseases in medicine. However, there has been a growing and substantiated interest in integrating quantum mechanics and quantum theory—both subsections of quantum physics—into medicine. Quantum physics is a branch of science that focuses on quantum mechanics and stems from quantum theory; quantum mechanics is the unique set of principles that explain the behavior of matter and energy that can in turn help explain quantum theory. Quantum physics is unique from most classical applications of physics in that it focuses on the physical properties of subatomic and fundamental particles, focusing on the micro-scale at the most extreme of levels, which classical physics does not achieve. New quantum technology and applications in the clinical sciences have demonstrated that understanding quantum mechanics can pose great benefits in learning more about cancer, telomere reduction, and the neurological framework of the human brain. In addition to this, applying quantum theory in medicine can allow for a much better understanding of DNA changes under mutagenesis, and can even help increase the rate at which diseases are diagnosed, even before symptoms arise in a patient. The intersectionality between the fields of quantum physics and medicine can facilitate a greater understanding of how certain diseases arise, and therefore help patients in future treatment. Hence, scientists in the field of medicine, as well as those in the field of quantum physics, have begun to realize that these two fields actually have more to do with one another than people usually realize, and it is worth delving into the intersectionality between these two fields and bringing them together as a means of advancing the field of medicine through unique applications in quantum theory.

## 2. Quantum Theory—Defining the Basics

Quantum theory, which is focused on the physical laws that govern structures such as the nucleus of an atom or even the subatomic particles within an atomic nucleus, has properties which make it unique to most other areas of physics. Quantum theory tends to give unique, discrete energy states by defining a wave function with given boundary conditions using Erwin Schrödinger’s wave equation [1], helping calculate and determine the specific energy states, amongst other specific quantities this wave function would be likely to have. Quantum theory disconnects itself from the normal classical theories of physics in that it does not treat variables along a continuous spectrum, but rather finds discrete states which these variables can be expected to have. It is interesting that with classical mechanics, one can usually find a definitive set of results and equations which exactly describe a particle’s motion. Meanwhile, in quantum mechanics, it is not exactly true that one will know where a particle may be. Rather, a statistical viewpoint is employed within quantum theory, focusing on the probabilities of where a particle could be found [2]. Together, these aspects of quantum theory and quantum mechanics make up what is known as quantum physics, encompassing this unique framework that analyzes the world with a new perspective. So, one may wonder how this subfield of physics could prove useful in helping advance the field of medicine. Current research has found underlying connections between certain quantum principles and certain properties of the human body.

## 3. Quantum Theory and the Mind

Certain research has delved into the consciousness of the human species as a means of understanding how our thought processes, thinking styles, and cognitive behavior relates to neuroanatomy and even in the goals of helping combat certain diseases. For instance, studies on the ability of human consciousness to adapt in different situations, such as under anesthesia during a surgery, or even studies on the general changes in consciousness when an individual begins to develop diseases such as Alzheimer’s disease, are extremely important because it can affect their decision-making skills and ability to act, even in a medical context [3]. Thus, learning how consciousness can be affected by drugs like anesthetics or even how it is affected by a neurodegenerative disease is extremely important in the field of medicine, as these can all affect a patient’s ability to perform daily tasks and to not feel pain during a medical procedure. However, research in the field of physics has found that classical mechanics is not constitutionally suited to accommodate consciousness based on certain mathematical characteristics, whereas quantum mechanics is. The nature of the information represented in the state of the brain, and the way this information enters the dynamics, is attributed to these mathematical characteristics [4]. Quantum mechanics is uniquely differentiable from classical mechanics in that, as previously described, the former relies on equations that help describe characteristics of a wave function and focuses on discrete probable states rather than a continuous spectrum. The mathematics employed in quantum physics is more akin to the statistical variety, whereas classical physics is more definitive and focused on exact, definitive answers for certain physical quantities. Neither is more right or wrong than the other; rather, they both just answer different types of questions. In the area of human consciousness, specifically with the ability of the brain to transmit signals and information and the structure of the neurological system of the human body, it has been found that the field of quantum theory actually holds great promise in helping us understand these underlying mechanisms.

Further research has focused on ways to use quantum mechanics as a means of describing certain decision-making processes and behaviors, which can help advance specific fields of medicine that deal with decision making and overall consciousness, such as in the brain with neurology and even with sedatives such as in anesthesiology, as described earlier. Physicists and mathematicians have tried relying on quantum-state reductions as a means of helping describe conscious interpretation by the human brain. The exact definition of a quantum-state reduction is that it “is the state change of the measured system caused by a measurement conditional upon the outcome of measurement, [and] is developed fully within quantum mechanics” [5]. In practical terms, however, the simple mechanism of these quantum-state reductions is to help describe the behavior of how things are inevitably perceived by conscious understanding. Quantum-state reductions are critical to understanding the human mind and consciousness because, as a conscious decision is determined and neurological networks transmit this information, it can be physically represented with wave function collapsing. These quantum-state reductions are critical because they can provide a physical understanding as to how neurological transmission occurs and how things are perceived by the consciousness, something which is extremely pertinent for neurology and the overall field of medicine. Described simply, “Consciousness and reality are related through the ‘measurement problem’ in quantum mechanics, i.e., why we do not consciously perceive particles as quantum superpositions of multiple possibilities, as they appear to be when unobserved, but rather perceive them consciously as being in definite states or locations” [6]. Mathematically, the goal of quantum-state reductions is to collapse the wave function or take a multitude of differing eigenstates and produce only one final eigenstate after there is some interaction or measurement with the external environment.

When analyzing the quantum theory behind this proposal, John von Neumann argued that one could use a projection postulate to help describe these so-called measurements, treating them as instantaneous and irreversible actions [7], using the following quantum formulization to describe how the wave function collapses (or in general, how a singular definitive conscious interpretation is reached in the brain with its final eigenstate):

(1) $$p->p\left(t\right)=U_{t}p{U_{t}}^{+}=e^{-\left(\frac{i}{\hbar }\right)Ht}pe^{\left(\frac{i}{\hbar }\right)Ht}{{}_{}}^{}$$

where “*H*” is the Hamiltonian operator, “*p*” is the state of the system before measurement, and “$U_{t}$” is the unitary time evolution operator. Henry Stapp used the groundwork von Neumann provided for these quantum-state reductions and purported that the boundary between these observed and observing systems mathematically described through this quantum representation is actually located in the human brain, and even further argued that quantum uncertainties at the level of the synapse can actually generate effects that are big enough to allow for superpositions of macroscopic brain activity, which occurs at the level of neuronal assemblies. This posited theory by Staff, taking from the mathematical basis of von Neumann, could have enormous implications for the field of medicine. If one uses this quantum theory to describe how neuronal assembly occurs in relation to how conscious behavior reaches a singular final state when interacting with an external stimulus, this could help describe certain changes in neural activity when a patient develops a neurodegenerative disease like Alzheimer’s disease. Moreover, finding the underlying mechanisms behind how anesthetics can inhibit conscious activity can be mathematically mapped out using these quantum projections, analyzing the process by which the final eigenstate is reached once these drugs are administered into the human body. It is an extremely fascinating theory, but more concrete research, evidence, and further experimentation are needed before this quantum application can be medically applied. Nonetheless, this demonstrates that the field of quantum physics has a lot more to offer in the field of medicine than people may have initially expected.

In general, quantum-state reductions can be explained through von Neumann’s work on consciousness and neuronal assemblies. Von Neumann’s work explains the conceptual distinction between an observed and observing system. Defining a measured object system (I), a measuring instrument (II), and the brain of a human observer (III), von Neumann found that it makes no difference whether the boundary between the observed and observing systems is posited between I and (II and III) or between (I and II) and III for the result of measurements on (I). This result is extremely profound, as this means that it is not significant whether a detector or the human brain is ultimately referred to as the “observer”. When analyzing the so-called collapse of a wave function, von Neumann’s work reveals that these irreversible actions could possibly hold significance in explaining conscious decision-making processes. Conscious decision-making behavior is a process that could indeed be described through these quantum-state reductions, where wave functions collapse and results are irreversible, as opposed to the continuous and reversible changes in a system according to the Schrödinger equation [8]. In turn, incorporating quantum theory into the field of medicine, and in the field of neurology, can help us reach a better understanding of how neural networks change over time in individuals with neurodegenerative diseases like Alzheimer’s, where conscious decision-making processes can be altered or weakened.

**Figure 1:**
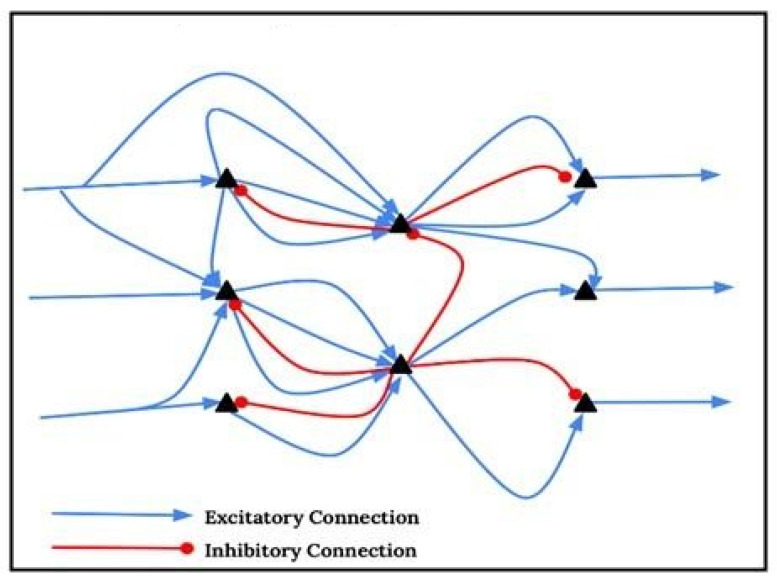
Equilibrium between neurons.

The neural assemblies of the brain can be described through both excitatory and inhibitory connections. A fair question to ask is whether there is a “neural correlate” for mental representation. In fact, the neural correlate of a mental representation can be characterized by the fact that the connectivity or coupling among those neurons form an assembly confined with respect to its environment, to which connectivity is weaker than within the assembly [8]. Thus, the neural correlation of a mental representation is activated if the neurons which form the assembly itself operate more actively by, e.g., producing higher firing rates than when in their normal mode. To maintain a stable operation of an activated neuronal assembly, there must be an equilibrium between the inhibitory and the excitatory connections amongst the neurons (see Figure 1). If the transfer function of individual neurons is strictly monotonic (which means increasing input leads to increasing output), assemblies will be difficult to stabilize, and equilibrium cannot be maintained. Therefore, the goal is to have results establishing a non-monotonic transfer function with a maximal output at intermediate input; this is of high significance for the modeling of neuronal assemblies [9]. Yet, mathematically, network models using lattices of coupled maps with a quadratic maximum are paradigmatic examples of this exact behavior, which is extremely beneficial [10]. Thus, incorporating these different network models, which are very similar to ideas described in quantum theory, where conscious decision making comes from collapsing wave functions and reaching one final irreversible state, can have great benefit in explaining certain biological phenomena, bringing an intersectionality between the two fields of quantum mechanics and medicine.

There are some concerns and controversies regarding applying quantum theory to the mind and neurological network transmissions. The general use of measurement that von Neumann describes—conscious decision making with collapsing wave functions—relies on certain principles that are in contention. The overall act of measurement is an essential aspect in the framework of quantum theory that has been the subject of controversy for more than eight decades now. In addition to this, there are other features of quantum theory which became attractive when discussing issues of consciousness, such as the concepts of complementarity and entanglement. Physicists such as Planck, Bohr, Schrödinger, and Pauli who helped lay the foundation for quantum theory focused on the various possible roles of quantum theory in reconsidering the “old conflict between physical determinism and conscious free will”. In turn, this new branch of physics did not necessarily always complement and agree with the previously utilized classical form of physics where energy was viewed as a continuous quantity. In turn, there is debate over whether fully relying on quantum theory to not only explain the neurological framework of the human body but also decision making is truly the most reliable approach. However, there is growing support and evidence as research continues to expand that demonstrates how the theories in the field of quantum physics do hold promise for helping us better understand how decision-making processes in the brain can be described.

## 4. Quantum Theory and Diagnosis

Another way in which the theories of quantum mechanics are beneficial for the field of medicine is that the ability to recognize the existence of a disease and diagnose a patient before it is symptomatically perceived can be enhanced through the application of quantum theory. For instance, there is a huge electrical field (~$10^{5}$ V/cm) on cellular membranes. This would mean that physically, this property of the membranes gives them the possibility to oscillate with eigen frequencies in the range of $10^{10}$ to $10^{11}$ Hz, and thus all cells of every living organism could possibly be looked at as an active center: the source of electromagnetic radiation [11]. Analyzing cellular membranes through this physical perspective applies a unique and novel way of thinking that can help us understand cellular biology through a different lens, understanding cellular structures from this physical standpoint. In particular, Sit’ko further proposed that a similar framework be applied to improve our understanding of the genome of an organism and how it acts in transcription and translation for proteins. It is known that all somatic cells of a specific organism have the same genome, or DNA. This, in conjunction with the previous assertion that each cell can be treated as an active center, allows for the idea that each coherence electromagnetic field within a cell can receive information from DNA regarding gene information. In turn, the genes which code for translation in proteins could be interpreted quantum mechanically as the transformation of a quasi-continuous spectrum of energy transitioning into a discrete one inherent to this specific organism only. This would mean that the formation of the spectrum of the characteristic eigenfrequencies is occurring. In other words, according to Sit’ko, certain cellular processes—such as translation—can be described as quantum mechanical transformations of energy, and thus can be explained through quantum mechanics. Sit’ko even went on to further conclude “that the disturbances of wave processes in meridians may appear long before the disease or its morpho-functional signs development, implying necessity for preventive action on this psychosomatic level of disorder” [11]. This implication would mean that using groundwork from quantum mechanics can actually help us detect diseases before they even become symptomatically present. Seeing disturbances in wave processes within the human body due to incoming diseases, despite requiring much more research, technological innovations, and further knowledge, is something that can readily advance detections in diseases and help save more lives in medicine.

Quantum theory also provides great explanations for well-isolated systems in terms of understanding scientific phenomena in the medical field. For instance, beginning with simple isolated quantum systems where both disorder and interactions are apparent, at a sufficiently high-level density, the stationary states become exceedingly complicated superpositions of simple quasiparticle excitations. At this stage, regularities typical of quantum chaos emerge and bring about signatures of thermalization. All the stages and the results of the processes leading to thermalization are explained using analytical and massive numerical examples for realistic atomic, nuclear, and spin systems, as well as for models with random parameters [12]. In other words, if a living biological system can be represented as a relatively well-isolated and cold system, quantum applications can be more easily applied, building up superpositions of different excitations to better describe the entire system as one entire holistic setting. This could be of great importance in areas like integrative medicine where it is essential to take multiple different aspects of the human body into account to have a better understanding of the diagnosis and treatment of a patient.

## 5. Quantum Theory and DNA

One other example of where incorporating quantum theory into medical applications would prove extremely helpful is with mutagenesis in DNA. Mutagenesis, or a mutation, is defined as a heritable change in the genetic material or composition of an organism [13]. Understanding the processes by which DNA undergoes mutations is extremely important because changes in DNA can lead to serious issues in the human body, such as development of normal cells into cancerous cells. It is even suggested that certain quantum theories could be applied to explain certain characteristics of mutagenesis. One important theory in quantum physics is the idea of superposition. Superposition is unique in that it “is a property of quantum particles to be in multiple places simultaneously within a zone of probability. This translates to a particle simultaneously occupying all possible locations, a property that is highly sought after to mimic in quantum computing as it would allow simultaneously testing of all outcomes” [14]. One may think that this does not have much to do with the areas of cellular biology and DNA or the genetic composition of an organism. However, that may not entirely be the case. Although the duration of superposition is debated, if certain processes occur on extremely small scales, then the effects regarding superposition could indeed become more prevalent. In the case of biologically important processes, such as with photosynthesis in a plant cell, these can occur at extremely small scales—in the range of femtoseconds to picoseconds. With mutagenesis, there is a possibility that superposition could play a role in this general process. Of course, to definitively determine this, more substantiated research regarding superposition would be required, as well as investigation into to what extent this quantum phenomenon is enhanced in mutagenesis, but this theory does have clear relevance to organisms and cellular biology in general. If one could accurately apply these principles of superposition to determining the causal factors behind certain types of mutations, this could help with curing certain cancers and helping treat patients. The one downside is that applications of superposition still require more research and advances in technology, but this quantum theory still proves to be promising for helping advance the future of medicine.

## 6. Quantum Theory and the Effects on Telomere Reduction

Another important medical application of quantum theory involves gaining an improved understanding of telomere reduction, which is known to have a role in cancerous cells. There is a very plausible hypothesis that quantum entropy may play a role in the cell-cycle checkpoints, which have to do with apoptosis in cancerous cells. Quantum entropy, also known as Von Neumann entropy, is similar to classical Gibbs entropy, but it is different in that it was developed under the realm of quantum mechanics based on utilizing an extended Hilbert space. In particular, there is evidence implying that when a reduction in telomere length induces a DNA damage response (which promotes senescence and inhibits cell checkpoints, which would normally induce apoptosis in cancer cells), quantum entropy conditions may affect the transpositional states, which can lead to alterations in cell-cycle checkpoints induced by higher energy demands in cells, leading to increased mitotic divisions [15]. When cells experience cancerous changes, they gain the ability to skip the M1 phase, and the suppression of cell checkpoints caused by the quantum entropy imbalance allows for this to happen. Within cancerous cells, there is an upregulation in the production of telomerase, the enzyme that adds length to telomeres through nucleotide additions to help promote continued cell division. When the telomerase activity is not great enough to overcome the energy demands of quantum-state entropy, which occurs due to bypassing the M1 phase, it inevitably leads to telomerases extending telomeres to allow ongoing cell proliferation in cancerous cells. In general, this hypothesis asserts that quantum entropy promotes changes to cell checkpoints during the crisis phase, which allows cancer cells to overtake normal cells through the manipulation of telomerase activity, leading to cancer in an organism [15]. Having a strong understanding of quantum entropy and analyzing how changes in quantum entropy can induce changes in cell-cycle checkpoints is extremely important. If quantum entropy could somehow be adjusted within a cell and help reverse the changes that occur to the cell-cycle checkpoint and hence telomerase activity, this could prove to be an extremely beneficial advancement in the field of medicine, helping us to treat cancer patients through quantum applications.

## 7. Macroscopic Quantum Phenomena in Medicine and Biology

The quantum holographic framework for macroscopic quantum phenomena can play a vital role in our understanding of more holistic aspects of medicine. A holistic approach to both integrative medicine and transpersonal psychology is essential because of their growing prominence in developed countries. Transpersonal psychology is an important branch of psychology that puts emphasis on human experiences that go beyond an individual’s ego or sense of personal self. Integrative medicine blends different forms of medicine into a comprehensive treatment plan, considering multiple aspects for treatments of care. Current research into psychosomatic diseases indicates the need to apply these holistic methods and a direct focus on acupuncture and human consciousness by applying macroscopic quantum theory [16]. For macroscopic quantum phenomena, there is a proposed quantum decoherence and quantum holographic (QDQH) approach to the conformational transitions of biomolecules [17]. This process cannot be interpreted kinetically based on semi-classical predictions and is known as the Levinthal paradox. The proposed solution of the Levinthal paradox provides a natural physical explanation with a series of processes for biomolecules, including chain folding and biomolecular recognition in an open biological environment. This biological process can be explained through quantum phenomena on a macroscopic level. It was found through intensive studies of these processes that fluctuations between non-complementary (approximately isolated) biomolecular states of energy and (environment-decoherence-selected non-isolated) biomolecular states of conformation are being repeated [18]. Hence, this QDQH approach allows the system of identical non-interacting and dynamically uncoupled proteins to be interpreted as a global spatial quantum ensemble of identical processors whose time-adapting density of conformational states can be presented as a cellular Hopfield-like quantum holographic neural network for an open-type cell. Therefore, applying the theory behind macroscopic quantum states can prove useful and beneficial in explaining certain biological processes, as explained with the Levinthal paradox. In terms of holistic medicine, the QDQH approach can even be extended to this type of clinical field. By incorporating macroscopic quantum phenomena into holistic medicine, our understanding of psychosomatic symptoms and therapy effectiveness can be improved through the aforementioned explanations of quantum energy states, dis-entanglement, and quantum holographic networks. Essential activities in the second (quantum holistic medicine) and third levels (conventional symptomatic medicine), neglecting the first level (different therapies), would result in further transpersonal transfer of memory attractors on the level of individual and collective consciousness in this and future generations. So, this implies it is necessary to focus on the origins of many psychosomatic problems at the underlying prenatal trans-generational level [16]. Hence, the QDQH approach can be used to help explain consciousness and psychosomatic symptoms and other pertinent aspects of holistic medicine. To conclude, utilizing macroscopic quantum phenomena will not only improve knowledge and understanding within the realm of holistic medicine, but it can also help expand upon further treatment for individuals in the future.

## 8. Conclusions

Overall, quantum physics can be integrated into a myriad of different medical applications. As well as in the realm of oncogenic processes, mutagenesis in DNA, and even the decision-making thought processes of the brain, quantum physics has an important role in even more medical matters. In the human body, even though it cannot be clearly seen, quantum mechanics is prevalent and has many functions and applications. Although quantum theories require more technological advancements and further research before their practical application can be achieved, there is clear evidence that quantum physics has an important role in the human body and can be used to help advance the field of medicine. Medicine is all about helping diagnose, treat, and cure patients with differing diseases and health-related issues. When we look at quantum medicine, we need to also consider the ethical concerns that arise from the implementation of quantum physics in medicine. We need to understand and investigate the implications and the effect it could have on patient care with regard to the aspects of privacy, consent, and equity. Incorporating quantum theory into the field of medicine can help bring new treatments to light and inspire new ways of thinking in this field, helping lead to innovative ideas that have never been thought of before in the world of healthcare. Currently, the technology necessary to implement quantum-physics-based approaches in clinical settings is still scarce. Even though technology is developing in different fields of medicine, at present, the idea of quantum physics being implemented is just that—an idea. One of the biggest challenges will be the sheer difference in the approach and learning curve that medical professionals and members of the health field need to overcome when new techniques like quantum-physics-based approaches become the standard of care. When we look at the medical field and clinical settings, one of the biggest concerns is the potential cost involved in creating and implementing quantum-based approaches. The advancements necessary to achieve quantum-based medical technologies are currently not available. Along with this, the application of quantum physics in medicine would necessitate costly infrastructure construction and training for medical professionals. The shift towards more quantum-physics-based medical technology is a novel concept, but achieving this will take many more years as there are too many practical challenges. As doctors continue to learn different techniques, styles, and methods to diagnose and treat patients, incorporating a strong understanding of quantum physics can help bridge the gap between different fields, and develop a strong intersectionality between different scientific disciplines, which is what modern medicine is. As beautifully explained in The Quantum Doctor: A Quantum Physicist Explains the Healing Power of Integral Medicine: “If any field needs integration, it is medicine. If any field needs an integrative paradigm that can make sense out of all the different models of healing, it is medicine” [19]. Hence, it’s time to go quantum in medicine.

## Data Availability

Not applicable.
